# Development of Post-Stroke Cognitive and Depressive Disturbances: Associations with Neurohumoral Indices

**DOI:** 10.3390/cimb44120429

**Published:** 2022-12-11

**Authors:** Marina Y. Zhanina, Tatyana A. Druzhkova, Alexander A. Yakovlev, Elena E. Vladimirova, Sofia V. Freiman, Natalia N. Eremina, Alla B. Guekht, Natalia V. Gulyaeva

**Affiliations:** 1 Research and Clinical Center for Neuropsychiatry of Moscow Healthcare Department, 115419 Moscow, Russia; 2 Institute of Higher Nervous Activity and Neurophysiology, Russian Academy of Sciences, 117485 Moscow, Russia; 3 M. P. Konchalovsky City Clinical Hospital, 124489 Moscow, Russia; 4 Department of Neurology, Neurosurgery and Medical Genetics, Pirogov Russian National Research Medical University, 119049 Moscow, Russia

**Keywords:** ischemic stroke, hypothalamic–pituitary–adrenal system, sympathoadrenal medullary system, interleukin 6, cortisol, post-stroke depressive disorder, post-stroke cognitive impairment

## Abstract

Neuropsychiatric complications, in particular cognitive and depressive disorders, are common consequences of ischemic stroke (IS) and complicate the rehabilitation, quality of life, and social adaptation of patients. The hypothalamic–pituitary–adrenal (HPA) system, sympathoadrenal medullary system (SAMS), and inflammatory processes are believed to be involved in the pathogenesis of these disorders. This study aimed to explore these systems in IS patients, including those with post-stroke cognitive and depressive disorders, within a year after IS. Indices of the HPA axis, inflammatory system, and SAMS were measured in blood serum (cortisol, interleukin-6 (IL-6)), plasma (adrenocorticotropic hormone), and saliva (cortisol, α-amylase). During one year after mild/moderate IS (NIHSS score 5.9 ± 4.3), serum cortisol and salivary α-amylase levels remained elevated in the total cohort. In the group with further cognitive decline, serum and salivary cortisol levels were elevated during the acute period of IS. In the group with poststroke depressive disorder, salivary α-amylase was constantly elevated, while serum IL-6 was minimal during the acute period. The results suggest prolonged hyperactivation of the HPA axis and SAMS after IS. Specifically, post-stroke cognitive impairment was associated with hyperactivation of the HPA axis during the acute IS period, while post-stroke depressive disorder was associated with the chronic inflammatory process and hyperactivation of SAMS during the follow-up period.

## 1. Introduction

Ischemic stroke (IS) remains one of the most critical medical and social problems due to high morbidity, high mortality rate, and subsequent persistent disability of patients [1]. An equally important medical and social problem is the set of neuropsychiatric complications, including post-stroke cognitive and depressive disorders [2,3]. These disorders often develop after stroke, are frequently comorbid, and significantly complicate the rehabilitation, everyday life, and social adaptation of patients, significantly reducing their quality of life.

Post-stroke cognitive impairment (PSCI) occurs in about one third of patients in the first month after a stroke [4] and persists for at least six months [5,6]. Changes in population demography, in particular, as well as increased life expectancy and improved survival after stroke have stimulated an increase in the absolute number of people with PSCI. Yet, despite the urgency of the problem, effective therapeutic approaches for PSCI prevention have not been developed so far, and revealing the risk factors of PSCI remains a live issue. PSCI is often comorbid with a post-stroke depressive disorder (PSDD) [7] developing in about 30% of patients within five years after a stroke [8,9]. This complicates the diagnosis and obstructs the choice of a subsequent adequate treatment [10].

In spite of extensive studies, the etiology and pathogenesis of PSCI and PSDD remain rather obscure. So far, no reliable correlation between the severity of focal brain injury and the probability of developing PSCI and PSDD has been found [11]. It has been hypothesized that focal brain injury results in uncontrolled activation of proinflammatory processes, inducing the disruption of the hypothalamic–pituitary–adrenal (HPA) axis control and hyperactivation of the HPA axis. The increased production of HPA hormones results in alterations in the expression and properties of corticoid receptors in key brain structures, primarily the limbic system, thus changing the regulatory mechanisms of the whole system controlled by negative feedback. Collectively, this leads to pathological functional and morphological impairments of most stress-sensitive brain areas—first and foremost the hippocampus with a high density of glucocorticoid receptors [12]. The hippocampus is a key structure involved in the formation of both memory and emotions, and its damage contributes to the occurrence of both cognitive and depressive disorders [13]. Besides the HPA axis, another leading neuroendocrine axis responsible for adaptation to stress is the sympathoadrenal medullary system (SAMS). The time course of α-amylase activity in the saliva is routinely used as a non-invasive index of SAMS activation [14]. Surprisingly, the extent and specific mechanisms of SAMS involvement in the development of PSCI and PSDD, as well as its interaction with the HPA system in the development of these post-stroke disorders remain virtually unexplored.

Thus, a comparative and comprehensive analysis of the status of HPA, SAMS, proinflammatory systems, and clinical parameters of patients performed at different post-stroke periods can provide important information on the involvement of these systems in the development of PSCI and PSDD. Potentially, such a study can reveal interrelations between these systems and contribute to developing approaches for the assessment of risks and prevention of these post-stroke disorders. The aims of this study were: (a) following the time course of changes in the biochemical indices of the HPA system, SAMS, and proinflammatory cytokine system in patients within a year of ischemic stroke (IS); (b) finding associations between these changes and the clinical parameters of the patients; and (c) assessing potential corticoid-dependent, neuroinflammation-mediated, and SAMS-related mechanisms of PSCI and PSDD development.

## 2. Materials and Methods

### 2.1. Subjects

Forty-five patients (33 men, 12 women, mean age 56 ± 5 years) hospitalized and treated at Konchalovsky Hospital of Moscow Health Care Department in 2019–2022 were selected for the study. For the control group, 32 healthy volunteers (19 men, 13 women, mean age 57 ± 4 years) were recruited.

The inclusion criteria for the IS patients were: age 45–80 years; ischemic cerebral infarction of hemispheric localization not involving limbic structures; mild to moderate stroke severity (1–15 points on the US National Institute of Health Stroke Severity Scale (NIHSS); admission to the hospital not later than 48 h after the CI. The exclusion criteria for all study participants were: previous stroke; craniocerebral injury with residual focal changes on CT/MRI or accompanied by loss of consciousness in the history; cognitive and depressive disorders in the history; presence of acute or chronic somatic and hormonal diseases; presence of alcohol or drug addiction.

The patients received therapy aimed at lowering blood pressure (Amlodipine, Enalapril, Moxonidine, Bisoprolol, Losartan); aimed at restoring blood flow in the vessel due to the dissolution of the thrombus inside the vascular bed (Actilyse, Aspirin); they were also treated with antioxidants (Mexidol), nootropics (Neipilept, Cerebrolysin), vasodilators (Magnesium sulfate), and antiarrhythmic drugs (Atorvastatin).

Socio-demographic information and medical and life history were collected from patients after stroke during the initial hospitalization. The focus of ischemic brain lesions was also determined by CT or MRI in all patients on admission.

Informed consent to participate in the study was signed by each included subject.

Clinical and laboratory examinations were performed:

- For 45 patients (33 men/12 women) in the acute period after stroke;
- For 41 patients (32 men/9 women) in the acute period and 30 days after stroke;
- For 33 patients (27 men/6 women) in the acute period, 30 days, and 180 days after stroke;
- For 29 patients (24 men/5 women) in the acute period, 30 days, 180 days, and 365 days after stroke;
- For control group participants (HC).

This study adhered to the tenets of the Declaration of Helsinki and had local ethics committee approval (#42, 23 August 2019) with informed consent obtained from all subjects.

### 2.2. Clinical Examination

Clinical examination included the evaluation of neurological, psychiatric, and cognitive parameters of the subjects by a neurologist and neuropsychologist based on the patient’s complaints, information obtained from third parties, and the results of the following neurological and psychometric tests:

The National Institutes of Health Stroke Scale, NIHSS—to assess the neurological status [15];

Montreal Cognitive Assessment, MoCA—to assess the cognitive status [16];

Hospital Anxiety and Depression Scale, HADS—to assess the psychoemotional status [17];

Beck Depression Inventory, BDI—to assess the depressive states [18];

Hamilton Rating Scale for Depression, HAM—to assess the depressive states [19];

Perceived Stress Scale, PSS—to assess the level of perceived stress [20];

The Holmes and Rahe Stress Inventory, or Social Readjustment Rating Scale, SRRS—to assess the stress level [21].

Modified Rankine Scale, mRS—to assess the degree of disability after a stroke [22].

Barthel Index for Activities of Daily Living, ADL—to assess daily life activity [23].

Stroop test—to assess the delay in reaction time between congruent and incongruent stimuli [24].

Luria memory words test—to assess different aspects of executive function, including the execution of a learned motor program, inhibitory control, attentional flexibility, working memory, and motor planning [25].

Head’s probe—to assess the spatial organization of movements [26].

Rey–Osterrieth complex figure test—to assess the visio-constructional ability and visual memory of neuropsychiatric disorders, including copying and recall tests [27].

The acoustic gnosis tests—to assess the perception of rhythms and the recognition of nonspeech sounds [28].

### 2.3. Laboratory Examination

#### 2.3.1. Serum and Blood Plasma

Blood sampling in patients at all examination periods, as well as in participants of the control group, was performed from the ulnar vein in the morning hours on an empty stomach into vacuum tubes with a S-Monovettec clotting activator to obtain blood serum and into vacuum tubes with S-Monovettec K3 EDTA to obtain blood plasma followed by centrifugation at 2000× *g* for 15 min at 4 °C.

The laboratory study included: the determination of baseline parameters of lipid, carbohydrate, and protein metabolism; assessment of adrenocorticotropic hormone (ACTH) level in blood plasma by enzyme immunoassay using ACTH ELISA Kits (Biomerica, Inc., Irvine, CA, USA); assessment of cortisol and interleukin-6 (IL-6) levels in blood serum by enzymatically amplified chemiluminescence on an Access2 immunochemical analyzer (Beckman Coulter, Brea, CA, USA) with appropriate kits (Beckman Coulter, Brea, CA, USA).

#### 2.3.2. Saliva

Saliva samples for cortisol and α-amylase assessment were collected in SaliCap low-adhesion tubes (IBL, Stockholm, Sweden) in an amount of approximately 1 mL, in the afternoon between 14:30 and 15:30 in order to minimize cortisol fluctuations. The procedure began at least 90 min after drug and food intake, and the oral cavity was rinsed thoroughly with water no later than 10 min before sampling. None of the participants had inflammatory or other visible changes in the oral cavity. All patients had a sufficient cognitive level to perform the procedure. Colored and cloudy saliva samples were discarded.

Saliva samples were centrifuged at 2000× *g* for 15 min. The supernatant fraction was collected in a clean test tube. Cortisol concentration was measured by enzymatically amplified chemiluminescence on an Access2 immunochemical analyzer (Beckman Coulter, Brea, CA, USA) using an appropriate kit (Beckman Coulter, Brea, CA, USA).

Before determining α-amylase activity, saliva samples were diluted 400-fold with sodium phosphate buffer (PBS, pH 7.2). The activity of α-amylase was measured using the kinetic colorimetric method on an ILAB Aries biochemical analyzer (Instrumentation Laboratory, Bedford, MA, USA).

#### 2.3.3. Hair Cortisol

Hair samples were collected at the posterior vertex of the scalp by cutting the hair as close as possible to the skin in the first days and 30 days after stroke. Hair samples were stored in separate airtight plastic containers until extraction. One centimeter of hair closest to the root was used for the analysis, corresponding to approximately 1 month prior to the study. The extraction and assessment of cortisol were performed as described in [29]. Cortisol measurements were performed using the Access cortisol kit (Beckman Coulter, Brea, CA, USA) for immunoassay for competitive interaction, in accordance with instructions provided by the manufacturer. Samples were analyzed using the ACCESS^®^ 2 automatic immunoassay analyzer (Beckman Coulter, Brea, CA, USA).

### 2.4. Statistical Analysis

Statistical analysis was performed using STATISTICA 10.0 (StatSoft Inc., Tulsa, OK USA) and GraphPad Prism version 9.4.1. software (GraphPad Software, Inc., San Diego, CA, USA). The normality of distribution was determined using the Shapiro–Wilk test. Fisher’s exact test was performed and Student’s *t*-test was used to compare two unrelated samples. The results are plotted as median with quartiles. The statistical significance of differences between unrelated samples with a non-normal distribution was determined using the Kruskal–Wallis test, followed by post hoc analysis (Dunn’s test). To analyze the statistical significance of differences between unrelated samples with normal distributions, a one-factor analysis of variance (ANOVA) followed by post hoc analysis (Tukey test) were used. For related samples, repeated measures analysis of variance (RM-ANOVA) or mixed analysis of variance (mixed-effects model) followed by post hoc analysis (Tukey or Sidak test) were used. Differences were considered significant at *p* < 0.05. A backward logistic regression model was used to quantify associations between the assumed predictor variables and this binominal outcome variable in either of the post-stroke depression or post-stroke cognitive decline components. The significance level for each variable’s entry to the model was set at 0.05. A logistic regression model involves some independent (predictor) variables (nominal or continuous) that may be used to predict a dependent (outcome) binominal variable. The input variables for both models included age, patient gender, parameters of neuropsychiatric scales as well as biochemical parameters.

## 3. Results

### 3.1. Time Course of Laboratory Markers after IS: Comparison with HC

The patients included in the study had a mild/moderate IS (NIHSS score 5.9 ± 4.3) with predominant localization in the middle cerebral artery (73%). Stroke with right-sided lateralization was detected in 49% of patients, with left-sided lateralization in 51%. Dyslipidemia was diagnosed in 27%, arrhythmia in 9%, diabetes mellitus in 11%, and arterial hypertension in 85% of patients. Six months after stroke, 58% of patients were diagnosed with cognitive and/or depressive disorders, which persisted until the end of the study period.

A comparison of laboratory markers of HPA (blood plasma adrenocorticotropic hormone (ACTH), salivary cortisol), SAMS (salivary α-amylase), proinflammatory cytokine system (interleukin-6, IL-6), evaluated at different stages in patients after IS was performed with healthy subjects (healthy control, HC) of similar age (*p* = 0.8), gender (*p* = 0.2), and sociodemographic characteristics.

**Figure 1:**
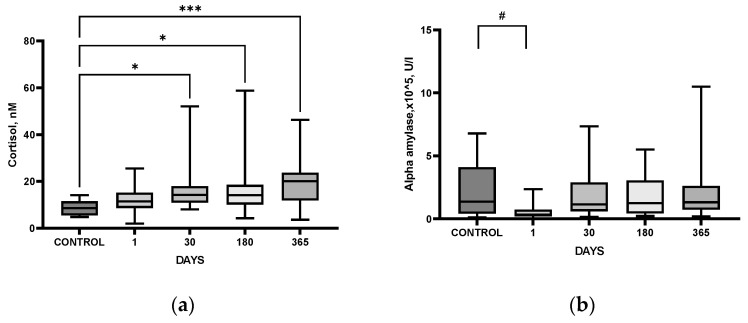

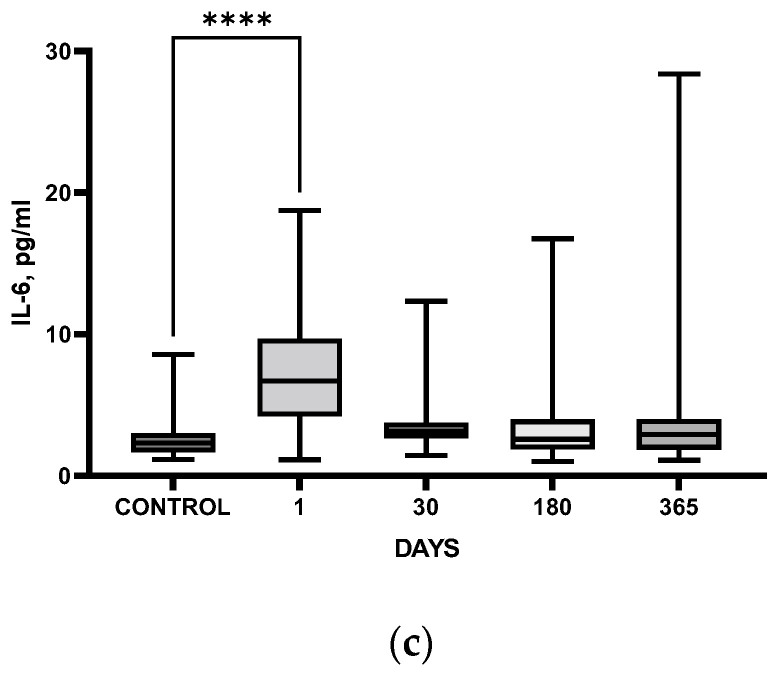
Time course of salivary cortisol (**a**), α-amylase (**b**), and serum IL-6 (**c**) within one year in IS patients and HC (CONTROL) group. Statistical differences between groups were assessed by Kruskal–Wallis ANOVA followed by post hoc Dunn’s test. # *p* < 0.1, * *p* < 0.05, *** *p* < 0.001, **** *p* < 0.0001. Kruskal–Wallis ANOVA: (**a**): *p* = 0.0002; (**b**): *p* = 0.0003, (**c**): *p* = 0.0001. HC, *n* = 32; patients after IS day 1, *n* = 45; day 30, *n* = 41; day 180, *n* = 33; day 365, *n* = 29.

It should be noted that most of the blood cortisol is protein-bound, mostly with corticoid-binding globulin (80%) and albumin (10%). The remaining 10% represents a small portion of free unbound blood cortisol in its biologically active form. However, since only the free form of cortisol is able to permeate the saliva, the measurement of salivary cortisol is considered a more reliable alternative for measuring cortisol in blood serum [15]. In the present study, cortisol levels in saliva were significantly higher in post-stroke patients as compared to those in HC at all follow-up time points, starting from day 30 after IS and reaching their maximal levels one year after IS (Figure 1a).

The activity of α-amylase in the saliva of patients, a noninvasive indicator of SAMS activity, was lower during the acute period after IS (a trend, *p* = 0.06) as compared to corresponding values in the HC group (Figure 1b). Serum IL-6 level in IS patients was significantly higher only in the acute period after IS as compared with the HC group (Figure 1c).

### 3.2. Time Course of Clinical Indices and Laboratory Markers after IS

**Figure 2:**
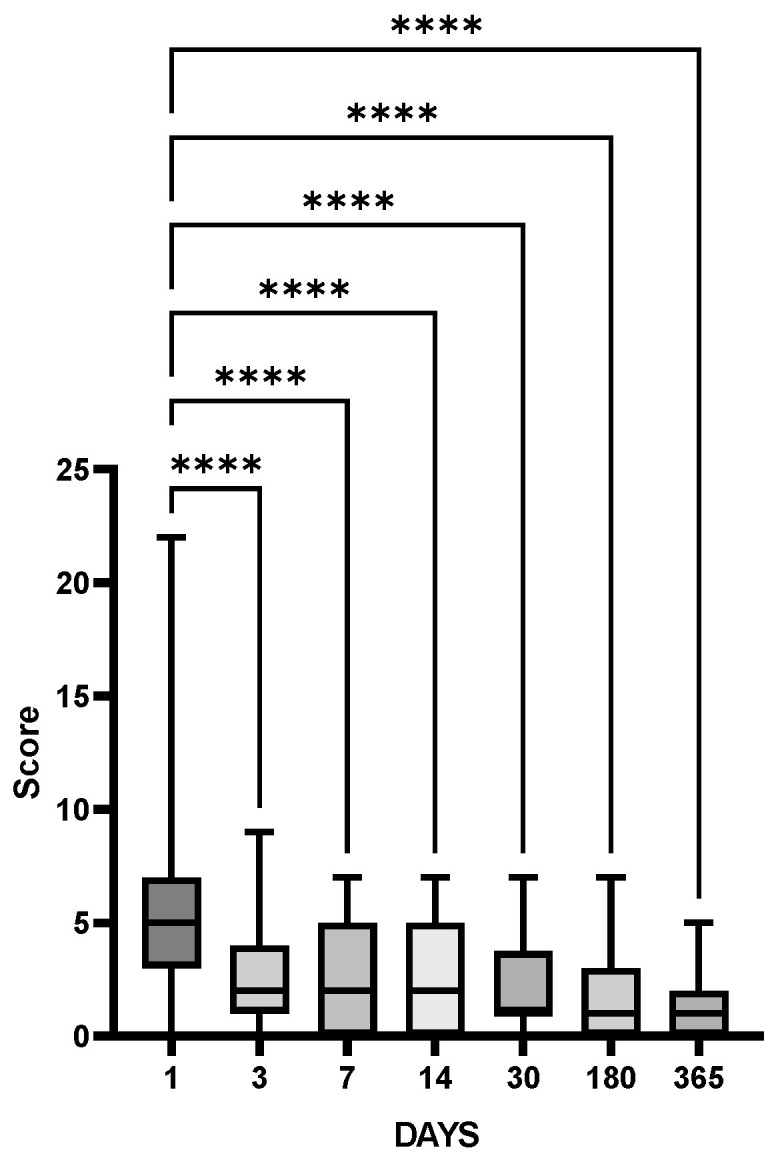
Neurological deficit according to the NIHSS scale within one year after IS. Statistical differences between groups were assessed by one-way ANOVA (F (6, 285) =15.53, *p* = 0.0001) followed by the post hoc Tukey test. **** *p* < 0.0001. Patients after IS day 1, *n* = 45; day 30, *n* = 41, day 180, *n* = 33; day 365, *n* = 29.

Clinical examination (assessment by a neurologist, NIHSS) demonstrated that neurological deficits in patients after IS completely recovered soon after IS and NIHSS scores remained low within a year (Figure 2).

Analyzing changes in laboratory markers after IS we should keep in mind that there is no “zero” time point in IS patients. The first available time point, 1 day, reflects the acute period corresponding to a definite point of the respective stress–response curve. The second point, 30 days, may reflect either nominal normalization or chronic changes in the level of the given index. 

**Figure 3:**
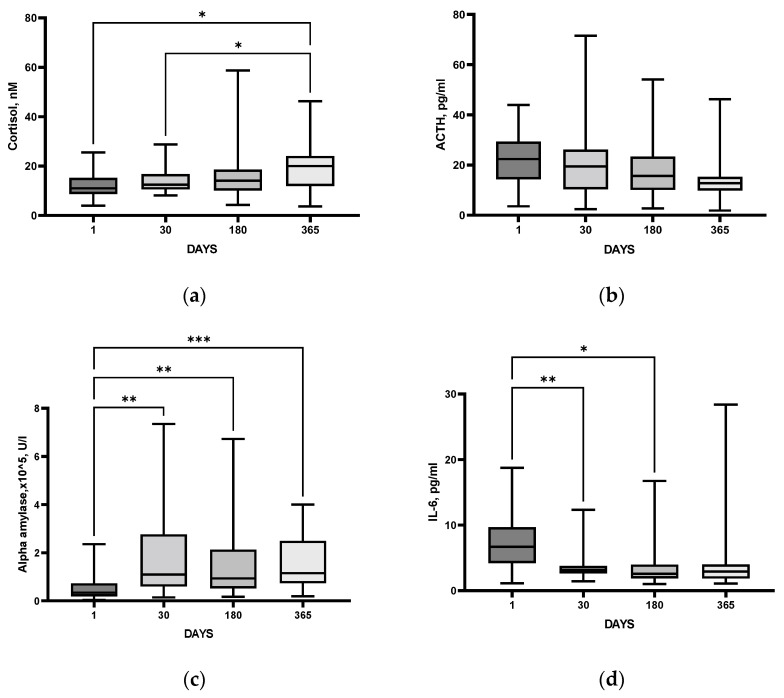
Time course of salivary cortisol (**a**), plasma ACTH (**b**), α-amylase (**c**), and serum IL-6 (**d**) within one year after IS. Statistical differences between groups were assessed by RM-ANOVA or by mixed-effects model ANOVA ((**a**): F (1.476, 36.89) = 5.352, *p* = 0.02; (**b**): F (1.919, 34.54) = 2.188, *p* = 0.13; (**c**): F (2.102, 48.35) = 7.659, *p* = 0.001; (**d**): F (2.393, 56.65) = 4.855, *p* = 0.008) followed by post hoc Tukey test. * *p* < 0.05, ** *p* < 0.01, *** *p* < 0.001.

Salivary cortisol did not decrease after the acute period; on the contrary, it increased one year after the IS (Figure 3a). However, plasma ACTH level did not change significantly (*p* = 0.13) at either time point after IS (Figure 3b). Such imbalance may be indicative of a negative feedback impairment in the HPA system after IS [11]. 

The salivary α-amylase activity demonstrated a significant and stable increase throughout the year as compared to the acute period. This may be indicative either of a persistent activation of SAMS during the post-stroke year, or of a decrease in SAMS functioning 1 day after IS (Figure 3c). Serum IL-6 levels, maximal in the acute period, decreased, showing a steady level during the year after SI (Figure 3d).

Thus, the time course of neurological deficit was similar to that of serum IL6, showing apparent normalization of the status with time, while both salivary cortisol and α-amylase demonstrated long-lasting increase as compared with respective acute values. 

Hair cortisol level was 48.7 ± 5.7 pg/mg on admission and 40.5 ± 3.6 pg/mg 30 days post-stroke (Mean ± SEM; *p* = 0.059, *t*-test) showing a decreasing trend after acute IS.

### 3.3. Time Course of Clinical Indices and Laboratory Markers after IS in Patients with and without Cognitive Impairment (PSCI)

According to the results of clinical examination, some patients demonstrated persistent cognitive impairment (opto-spatial agnosia and spatial apraxia, reduced attention and memory, lack of criticism, and regulatory dyspraxia) as early as day 14 after IS. To assess the potential involvement of each system (HPA, SAMS, and proinflammatory cytokines) in the development of PSCI, IS patients were divided into two groups: with detected cognitive impairment and without cognitive impairment. 

Comparative characteristics of sociodemographic and clinical parameters, as well as data from psychometric scales for the groups of patients with and without PSCI, are shown in Table S1. 

**Figure 4:**
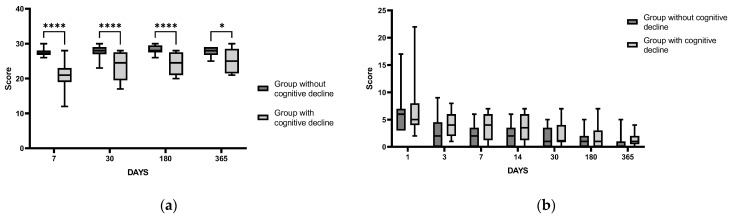
Time course of MoCA scores (**a**) and NIHSS (**b**) in IS patients with and without PSCI. Differences between groups were assessed by RM-ANOVA or by mixed-effects model ANOVA ((**a**): F (3, 87) = 5.807, *p* = 0.001; (**b**): F (6, 177) = 0.9638, *p* = 0.45) followed by the post hoc Sidak test. MoCA scores for groups without (*n* = 17) and with PSCI (*n* = 16), *p* = 0.0012. NIHSS for groups without (*n* = 17) and with PSCI (*n* = 16), *p* = 0.5. * *p* < 0.05, **** *p* < 0.0001.

It should be noted that the significant difference between the groups of patients with and without PSCI according to the MoCA scale revealed 7 days after IS was maintained throughout the whole study period (Figure 4a), indicating a persistent change in the cognitive status of this fraction of IS patients. However, the scores of the NIHSS reflecting the neurological status of patients did not differ significantly between the groups with and without PSCI throughout the study period (Figure 4b), indicating the apparent absence of a significant association between neurological status and cognitive impairment after IS in the cohort of patients in our study.

**Figure 5:**
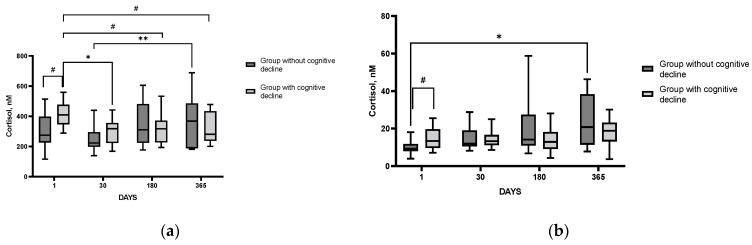
Changes in cortisol levels in blood serum (**a**) and saliva (**b**) in the groups of patients with and without cognitive impairment. Statistical differences between groups were assessed by RM-ANOVA or by mixed-effects model ANOVA ((**a**): F (3, 60) = 3.876, *p* = 0.013; (**b**): F (3, 71) = 2.464, *p* = 0.07) followed by post hoc analysis (Tukey test). # *p* < 0.1, * *p* < 0.05, ** *p* < 0.01. Group without cognitive decline (*n* = 11); group with cognitive decline (*n* = 11).

Analysis of the time course of changes in laboratory indices indicative of SAMS activity (salivary α-amylase) and inflammation (IL-6) did not reveal statistically significant differences between the groups of patients with and without PSCI (*p* = 0.66, 0.35, respectively) throughout the study period. However, significant changes between the groups were found when assessing the time course of changes in cortisol levels in blood serum (*p* = 0.013) and saliva (*p* = 0.07). Serum cortisol and saliva levels were higher in patients with PSCI in the acute period of IS as compared to the corresponding parameters in patients without cognitive impairment (Figure 5a,b). Yet, no significant differences between the groups were found in the time course of ACTH levels in blood plasma (*p* = 0.53). The results indicate that in the acute IS period, patients with detected cognitive impairment demonstrate a higher degree of HPA hyperactivation as compared to patients without such impairment.

The logistic regression showed that an increase of salivary cortisol by 1 nmol/L significantly increased the odds ratio for PSCI 1.42 times while each year increased the odds ratio 1.13 times (Table S3).

### 3.4. Time Course of Changes in Clinical and Laboratory Parameters after IS in Patients with and without Depressive Disorder

On the 30th day after the IS, 30% of patients who underwent clinical examination were diagnosed with PSDD. To assess the contribution of each system (HPA, SAMS, and proinflammatory cytokines) in the development of PSDD, patients were divided into two groups: with and without a detected depressive disorder.

The comparative characteristics of sociodemographic and clinical parameters, as well as the psychometric scales data for the groups of patients with and without PSDD, are shown in Table S2.

**Figure 6:**
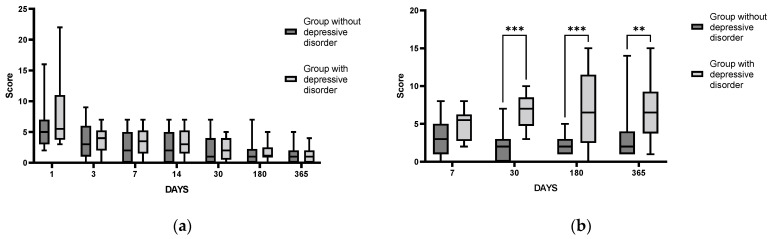
Time course of NIHSS (a) and HADS scores (b) in patient groups with and without PSDD. NIHSS scale for groups without depressive disorder (*n* = 23) and with depressive disorder (*n* = 10) *p* = 0.6; HADS scale for the group without depressive disorder (*n* = 23) and with depressive disorder (*n* = 10) *p* = 0.04. Statistical differences between groups were assessed by RM-ANOVA or by mixed-effects model ANOVA ((**a**): F (6, 177) = 0.8, *p* = 0.57; (**b**): F (3, 87) = 2.844, *p* = 0.04) followed by post hoc analysis (Sidak test). ** *p* < 0.01, *** *p* < 0.001.

The scores on the NIHSS scale evaluating the neurological states of patients in both groups did not significantly differ throughout the study period (Figure 6a), which indirectly indicates no significant influence of neurological status on the occurrence of PSDD in patients after IS. However, significant differences in the HADS (Figure 6b), HAM, BDI, PSS, and mRS scales between the groups of patients with and without PSDD detected one month after the IS, persisted throughout the entire follow-up period. This is indicative of stable changes in their psychoemotional status throughout the post-stroke year.

In contrast to the data obtained when comparing the groups of patients with and without PSCI, a comparative analysis of the HPA indices’ time course after IS did not yield reliable differences. Comparing the groups of patients with and without PSDD, we found significant differences neither in the changes in salivary nor serum cortisol levels, nor ACTH in plasma (*p* = 0.75; 0.5; 0.9, respectively).

**Figure 7:**
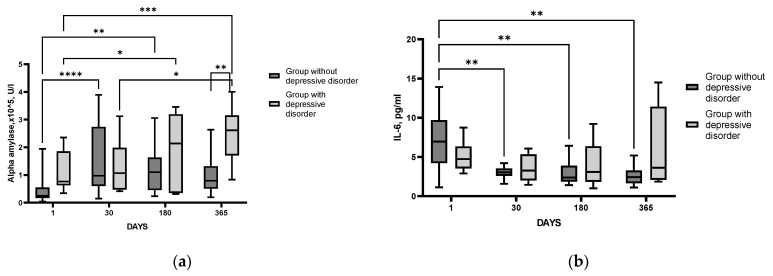
Time course of α-amylase in saliva (**a**) and IL-6 in blood serum (**b**) in groups of patients with and without PSDD. Statistical differences between groups were assessed by RM-ANOVA or mixed-effects model ANOVA ((**a**): F (3, 64) = 4.087, *p* = 0.01; (**b**): F (3, 62) = 3.789, *p* = 0.02) followed by post hoc analysis (Tukey test). * *p* < 0.05, ** *p* < 0.01,*** *p* < 0.001, **** *p* < 0.0001. Group with PSDD disorder (*n* = 9); group without PSDD (*n* = 21).

In the group of patients with detected PSDD, the level of α-amylase activity in saliva remained statistically significantly increased throughout the study period, staying stably high one year after IS. In the group of patients without detected PSDD, the level of α-amylase activity was increased only for the first six months after stroke, with subsequent normalization of the parameter (Figure 7a). Thus, we can assume that in patients with diagnosed PSDD, SAMS hyperactivation was more prolonged as compared with patients without PSDD.

A comparative analysis of proinflammatory system state in the groups of patients with and without PSDD, with IL-6 as a key indicator, showed an important difference between the groups. IL-6 levels in patients without PSDD were elevated only in the acute period and “normalized” as early as day 30 after IS. By contrast, in patients diagnosed with PSDD, the level of IL-6 did not change significantly during the whole follow-up period (Figure 7b). Based on these results, we can assume that patients with diagnosed PSDD had impaired reactivity of the proinflammatory cytokine system as compared with post-stroke patients without this disorder. The logistic regression could not reveal significant predictors for PSDD in the cohort of patients studied.

Unfortunately, it was not possible to analyze the time course of changes in laboratory markers reflecting HPA and SAMS functioning, as well as IL-6 in the group of patients with comorbid PSCI and PSDD due to the small number of patients in this group. Therefore, these data were not included in the analysis.

## 4. Discussion

Before discussing the data, it should be clearly understood that the first available point in patients after IS is 1 day, the acute period. This means that we do not have (and cannot have in any case) a “zero point”, a conventional “background” for either patient or index. This fact significantly affects the interpretation of the time course data, making it more presumptive. We also can neither exclude nor verify that in the patients who developed PSCI or PSDD some indices might be initially abnormal. This is an obvious though insoluble limitation of the study.

### 4.1. Delayed Post-Stroke Cognitive and Emotional Disturbances

In addition to immediate and long-lasting neurological deficits, IS can be followed by the development of delayed cognitive and emotional disturbances. Progress in the treatment and prevention of delayed consequences of IS has been frustratingly slow, and the insufficient knowledge of their mechanisms is one of the reasons. Approximately two-thirds of all middle-aged and elderly stroke patients develop cognitive impairment, and one in three develops dementia [30]. Post-stroke depression, a relatively common complication of IS, occurs in a significant number of patients and, similarly to cognitive impairment, comprises an important complication of IS, leading to greater disability as well as increased mortality [31]. Despite the extensive literature on this topic, there is no agreement on the frequency or risk factors for post-stroke depression [32]. In our study, about half of IS patients developed PSCI, while about one-third demonstrated PSDD. The pathophysiology of post-stroke depression is multifactorial and probably involves alterations in the monoamine and neurotrophic system, increased inflammation with dysregulation of the HPA axis, and glutamate-mediated excitotoxicity [33]. 

### 4.2. Involvement of HPA Axis in IS consequences

The HPA axis dysfunction associated with stroke is generally neglected, though both clinical data and the results from rodent models of IS show that glucocorticoids are tightly involved in IS-induced brain dysfunction (see [34] for review). Adrenal glucocorticoid stress response in acute stroke is harmful. High cortisol levels are associated with poor outcomes and mortality of patients with stroke [35]. In patients with acute IS, high serum cortisol at admission correlated with clinical severity according to NIHSS, as well as poor prognosis and functional outcome evaluated by the modified Rankin Scale [36]. In the majority of studies, cortisol levels were high in the first week after IS, and higher cortisol was associated with dependency, morbidity, depression, and mortality [37], though single studies demonstrated that, although IS was associated with a change in serum cortisol level, this change had no prognostic value [38]. In our study, comparative analysis of patients after IS and HC group revealed significantly elevated cortisol levels during the year after IS, starting from day 30 (Figure 1a), and persisting during the follow-up period (Figure 3a), though no significant changes in ACTH levels could be detected (Figure 3b). The results indicate that despite the complete recovery of neurological deficit in the cohort studied (Figure 2), the patients had persistent disturbances in HPA functioning within a year after stroke.

Gyawali et al. [39] demonstrated that compared with age-matched controls, IS long-term survivors reported greater levels of perceived stress, and lower levels of resilience which were independently associated with stroke outcomes. In particular, these relationships were observed for cognitive outcomes including mood, memory, and communication. Ample control of the HPA axis is vital for a better prognosis after IS. Hypercortisolism is associated with cognitive dysfunction early after IS, while both high and low circulating cortisol levels are associated with increased mortality after IS [40]. Serum cortisol level was independently associated with cerebral small vessel disease burden and cognitive impairment [41]. A higher salivary cortisol level after IS indicated a higher probability of mild cognitive impairment occurrence, and the salivary cortisol level can be used as a predictive marker for MCI occurrence [42]. Here we showed that patients with higher cortisol levels in the acute period after the IS (Figure 5) subsequently developed cognitive impairment, which persisted throughout the entire follow-up period (Figure 4b). These results confirm the data of the Tel Aviv Brain Acute Stroke Cohort (TABASCO) study [43]. The involvement of stress-related endocrine dysregulation in the development of post-stroke cognitive changes was assessed in a long-term study of patients who suffered a first mild to moderate IS or transient ischemic attack; hair cortisol accumulation on hospital admission was evaluated as an indicator of cortisol levels during a month before IS. Hair cortisol is a retrospective measure of a previous time period based on the length of hair collected. Since hair grows approximately 1 cm/month, the 1 cm-long segment of hair analyzed in this study reflects cortisol accumulation within a month preceding IS [29]. High cortisol levels in hair were shown to be significantly associated with worse cognitive outcomes at 6, 12, and 24 months after stroke [43]. A trend toward augmented acute hair cortisol levels found in our study is consistent with increased stress levels preceding IS. However, we were unable to reveal any difference in this index between groups with and without PSCI or PSDD, most probably because of the rather small cohort of IS patients in our study. Later, the TABASCO group demonstrated that higher salivary cortisol levels immediately after IS were associated with greater subsequent cognitive deficits, brain atrophy, and white matter damage up to 24 months after stroke [44]. Patients with high salivary cortisol at baseline and throughout the study had worse memory compared to patients with low cortisol levels. A number of other studies have confirmed the relationship between elevated cortisol levels and impaired cognitive performance [45,46,47]. In our study, salivary cortisol during the acute post-stroke period and age were predictors of PSDD (Table S3)

The altered HPA functioning could induce fluctuations of cortisol levels and be associated with the onset of post-stroke depression [48], and the expression of serum cortisol level is believed to be closely correlated with the incidence of depression after IS [49]. Poststroke depression is closely related to a dysfunctional HPA axis indicated by blunted salivary cortisol awakening response [50]. Anhedonia in IS patients was associated with the volume of stroke lesions in the parahippocampal gyrus and with dysfunction of the HPA assessed by salivary cortisol levels [51]. However, in the present study, we failed to find significant differences in serum or salivary cortisol and plasma ACTH between patients with and without PSDD. 

### 4.3. Involvement of SAMS in IS Consequences

The functional activity of another important stress-related neuroendocrine system, the SAMS, is routinely accessed using changes in salivary α-amylase. It is well known that salivary α-amylase secretion is regulated by the autonomic nervous system, and its level increases with sympathetic nervous system activation in response to stress [14,52,53]. The levels of salivary α-amylase in stroke patients were not clearly investigated, but available results indicate that patients with stroke have higher salivary α-amylase activity compared to healthy subjects [54]. Changes in SAMS may be involved in the development of neuropsychiatric disorders, though the data are scarce and controversial [55,56]. It is suggested that while salivary cortisol can differentiate between healthy controls and patients with psychiatric disorders, salivary α-amylase may be a putative candidate biomarker for major depression; specifically, salivary α-amylase at awakening and not cortisol differentiates major depression from other psychiatric disorders in outpatients [57].

In the present study α-amylase activity in the saliva of patients after IS was increased on day 30 after SI as compared to the values during the acute period, and it stayed stable, remaining elevated for a year (Figure 3c). We can suggest that persistent activation of the SAMS detected during the follow-up period takes place in our cohort of patients after an IS. Importantly, the most pronounced and prolonged SAMS activation was evident in patients with diagnosed PSDD as compared to poststroke patients without identified depression (Figure 7a). It is believed that the level of α-amylase activity in saliva reflects noradrenaline changes [58]. Noradrenaline is synthesized in the locus coeruleus and enters various structures of the limbic system (amygdala, hippocampus, and hypothalamus), where it binds to α- and β-adrenoreceptors [59]. A number of authors associate the development of depressive disorder with changes in the density and sensitivity of α2a-adrenoreceptors in the brain, as well as to decreased levels of noradrenaline transporter in the locus coeruleus [60,61]. It can be assumed that the development of post-stroke depression involves a mechanism related to neurotransmission in SAMS.

### 4.4. Involvement of Inflammatory Response in IS Consequences

Researchers are increasingly interested in the inflammatory response, in particular, in the role of pro-inflammatory cytokines, triggered by cerebral ischemia, and their involvement in the consequences of IS [62]. Interleukins play multiple roles in IS through information transmission, activation, and regulation of immune cells, and thus impacting the outcome of IS [63]. It has been reported that compared to controls, IS patients have higher IL-6, IL-8, and TNFα protein in plasma and lower IL-6, IL-8, TNFα, IL-1α, and IL-1β mRNA in leukocytes within 72 h after stroke. However, only the elevation of IL-6 correlated with the severity and prognosis of stroke, and that of other cytokines in plasma proteins after IS appeared secondary to IL-6 [64]. Thus, IL-6 may be the key mediator of the circulating pro-inflammatory cytokines network. 

Though the association between inflammatory indices and cognitive decline in post-stroke situations remains obscure, recent data suggest an involvement of IL-6 in PSCI. Wang et al. showed that after IS or transient ischemic attacks, elevated IL-6 levels were independently associated with the reduction in MoCA [65]. In this study, post-stroke cognitive decline one year after IS was detected in about 24% of patients. Patients in the highest quartile of IL-6 level had a higher risk of cognitive decline than those in the first quartile, after adjusting for potential risk factors. Accumulating evidence suggests that pro-inflammatory cytokines, in particular peripheral IL-6, play an important role in the pathogenesis of depression [66]. Changes in cytokines have been hypothesized to be associated with the etiology of post-stroke depression, in particular. The IL-6 level and afternoon cortisol levels were elevated significantly in IS patients with PSDD [47]. The plasma level of IL-6, but not TNFα, sIL-6R, or IL-1ra, was higher in patients who developed depressive symptoms at 3 months after IS; plasma IL-6 predicted the severity of depressive symptoms at 3 months after stroke [67]. 

In the present study, using IL-6 as a reference cytokine, we have shown that the proinflammatory cytokine system is activated in patients in the acute period after IS as compared with the control group (Figure 1c). In IS patients, maximal IL-6 level was detected during the acute period, decreased by day 30, and remained stable during the whole follow-up period (Figure 3d). However, in patients with a PSDD, IL-6 levels did not change after the acute period, remaining constant, while in patients without a depressive disorder, this parameter decreased by 30 days after IS (Figure 7b). Our results confirm numerous data from clinical studies demonstrating increased levels of IL-6 in the blood of depressed patients [68]. Uncontrolled activation of the proinflammatory cytokine system occurs during brain damage, which causes disruption of the HPA axis functioning and may be a trigger mechanism for the development of PSDD [69]. In the present study, this is indirectly confirmed by the fact that the group of patients with PSDD, unlike post-stroke patients without a depressive disorder, had a higher Perceived Stress Scale (PSS) score (Table S2).

## 5. Conclusions

Changes in HPA axis indicators (serum and salivary cortisol) and SAMS (salivary α-amylase) during the year after moderate IS indicate disturbances in the functioning of these systems, particularly, hyperactivation. The progress of poststroke cognitive impairment is associated with hyperactivation of the HPA axis in the acute period after IS, while the development of poststroke depressive disorder is associated with a chronic inflammatory process and hyperactivation of SAMS during the follow-up period. Though logistic regression could not reveal significant predictors for PSDD in our cohort of patients, an increase in salivary cortisol and in age can predict the development of PSCI. An essential line of research in the future would be the assessment of neurohumoral indices and post-stroke cognitive and depressive disturbances for different types of stroke. In particular, this may be important for lacunar versus non-lacunar acute stroke, since the pathophysiology, prognosis, and clinical features of acute small-vessel ischemic strokes are different from other types of cerebral infarcts, lacunar infarcts demonstrating a better functional prognosis [70]. 

## 6. Limitations

One of the major limitations of this study is the rather small cohort of IS patients, especially at the end of the follow-up period. This limitation prevented us from analyzing the time course of laboratory markers reflecting HPA and SAMS functioning, as well as IL-6 in the group of patients with comorbid PSCI and PSDD which was too small for a decent statistical analysis. An unavoidable limitation of our prospective study design is the impossibility of having the baseline (before the stroke) laboratory data.

## Data Availability

All data generated or analyzed during this study are included in this published article. Primary datasets generated during and/or analyzed during the current study are available from the corresponding author upon reasonable request.

## Supplementary Materials

The following supporting information can be downloaded at: https://www.mdpi.com/article/10.3390/cimb44120429/s1, Table S1: Characteristics of patients with and without cognitive impairment; Table S2: Characteristics of patients with and without depressive disorder; Table S3: The input variables in the model that were recognized as being significant predictors of post-stroke cognitive decline in multiple logistic regression analysis.

## Abbreviations

ACTH, adrenocorticotropic hormone; ADL, Barthel Index for Activities of Daily Living; BDI, Beck Depression Inventory; HC, healthy control; HPA, hypothalamic-pituitary-adrenal; HAM, Hamilton Rating Scale for Depression; IL-6, interleukin-6; ischemic stroke, IS; HADS, Hospital Anxiety and Depression Scale; MoCA, Montreal Cognitive Assessment; mRS, Modified Rankine Scale; NIHSS, The National Institutes of Health Stroke Scale; PSCI, post-stroke cognitive impairment; PSDD, post-stroke depressive disorder; PSS, Perceived Stress Scale; SAMS, sympathoadrenal medullary system; SRRS, The Holmes and Rahe Stress Inventory, or Social Readjustment Rating Scale.
