# Supplementary material for: Development of Post-Stroke Cognitive and Depressive Disturbances: Associations with Neurohumoral Indices
Source: Curr Issues Mol Biol. 2022 Dec 11;44(12):6290–305. doi: 10.3390/cimb44120429 (PMC9776624; doi:10.3390/cimb44120429)
Supplement: Supplementary file 1 [file cimb-44-00429-s001.zip › cimb-2048264-supplementary.pdf]

Supplementary material

Development of Post-Stroke Cognitive and Depressive Disturbances: Associations with Neurohumoral Indices

Authors: Marina Y. Zhanina, Tatyana A. Druzhkova, Alexander A. Yakovlev, Elena E. Vladimirova, Sofia V. Freiman, Natalia N. Eremina, Alla B. Guekht and Natalia V. Gulyaeva

Table S1. Characteristics of patients with and without cognitive impairment.

Parameters	Patients with PSCI	Patients without PSCI	*p*-value
Gender (male/female), n	12/4	15/2	0.30
Age (M±σ), years	58±12	53±9	0.25
Education (middle/higher), %	87.5/12.5	64.7/35.3	0.13
Dyslipidemia, %	12.5	23.5	0.36
Arrhythmia, %	25	0	0.05
Diabetes, %	6	12	0.52
Smoking, %	44	47	0.56
Localization of IS in the middle cerebral artery, %	81	59	0.15
Lateralization of IS (right/left), %	50/50	70.6/29.4	0.20
NIHSS (M±σ), points	2.2±2.0	1.8±1.5	0.50
HAM scale (M±σ), points	6.0±5.5	3.0±3.5	0.06
BDI scale (M±σ), points	6.4±5.0	4.8±4.2	0.30
HADS scale (M±σ), points	4.4±3.5	3±2.8	0.20
MoCA scale (M±σ), points	23.3±4	27.6±2.0	0.0004***
SRRS scale (M±σ), points	150.4±55.6	179.3±89.5	0.96
PSS (M±σ), points	34.8±9.0	29.8±9.3	0.13
mRS (M±σ), points	0.5±0.5	0.1±0.3	0.02*
ADL scale (M±σ), points	99.4±1.7	99.4±1.7	1.00

Data of all psychometric and neurological scales are presented for the 30th day after IS, except of the SRRS, for which the data are presented for 180th day after SI. ***p<0.001.

Table S2. Characteristics of patients with and without depressive disorder.

Parameters	Patients with PSDD	Patients without PSDD	*p*-value
Gender (male/female), n	8/2	19/4	0.60
Age (M±σ), years	61±7.2	54±7	0.20
Education (middle/higher), %	60/40	74/26	0.34
Dyslipidemia, %	30	17.4	0.40
Arrhythmia, %	30	4.4	0.07
Diabetes, %	10	8.7	0.66
Smoking, %	40	48	0.49
Stroke localization in the middle cerebral artery, %	80	65	0.34
Stroke lateralization (right/left), %	40/60	70/30	0,11
NIHSS scale (M±σ), points	2.1±2.0	2.0±2.0	0.50
HAM scale (M±σ), points	8.7±6.0	2.7±3.0	0.0003***

BDI scale (M±σ), points	10.2±4.0	3.5±3.0	0.000007****
HADS scale (M±σ), points	6.8±2.3	2.3±2.5	0.00004****
MoCA scale (M±σ), points	25±5.0	26±3.4	0.70
SRRS scale (M±σ), points	192.7±101.5	152.1±57.3	0.20
PSS scale (M±σ), points	38.0±7.0	30.0±9.3	0.02*
mRS scale (M±σ), points	0.6±0.5	0.2±0.4	0.01*
ADL scale (M±σ), points	99.0±2.1	99.6±1.4	0.40

Data of all psychometric and neurological scales are presented for the 30th day after IS, except of the SRRS, for which the data are presented for 180th day after SI. ***p<0.001, ****p<0.0001.

Table S3. The input variables in the model that were recognized as being significant predictors of post-stroke cognitive decline in multiple logistic regression analysis.

	**B**	**SE**	***p*-Value**	**OR**	**95% CI**
Model of post-stroke cognitive decline					
Age	0.12127	0.05564	0.0293	1.13	1.02–1.28
Cortisol in saliva, day 1	0.34788	0.14766	0.0185	1.42	1.11–1.99
Constant	−10.74099	4.30531	0.0126	0.02	-

R square of post-stroke cognitive decline = 0.73. B, unstandardized regression coefficient; CI, confidence interval; SE, standard error, OR, odds ratio.
